# Variation in Aquaporin and Physiological Responses Among *Pinus contorta* Families Under Different Moisture Conditions

**DOI:** 10.3390/plants8010013

**Published:** 2019-01-06

**Authors:** Shanjida Khan, Barb R. Thomas, Raul de la Mata, Morgan J. Randall, Wenqing Zhang, Janusz J. Zwiazek

**Affiliations:** 1 Department of Renewable Resources, University of Alberta, 442 Earth Sciences Bldg., Edmonton, AB T6G 2E3, Canada; shanjida@ualberta.ca (S.K.); bthomas@ualberta.ca (B.R.T.); mrandall@ualberta.ca (M.J.R.); wenqing3@ualberta.ca (W.Z.); 2 Institut de Recerca i Tecnología Agroalimentàries (IRTA), Torre Marimon, 08140 Caldes de Montbui, Spain; delamatapombo@yahoo.es

**Keywords:** aquaporins, drought, *Pinus contorta*, transcript abundance, photosynthesis, stomatal conductance, water use efficiency

## Abstract

A population of eight open pollinated families of *Pinus contorta* was selected from sites varying in precipitation regimes and elevation to examine the possible role of aquaporins in adaptation to different moisture conditions. Five *Pinus contorta* aquaporins encoding *PiconPIP2;1*, *PiconPIP2;2*, *PiconPIP2;3*, *PiconPIP1;2*, and *PiconTIP1;1* were cloned and detailed structural analyses were conducted to provide essential information that can explain their biological and molecular function. All five PiconAQPs contained hydrophilic aromatic/arginine selective filters to facilitate the transport of water. Transcript abundance patterns of *PiconAQPs* varied significantly across the *P. contorta* families under varying soil moisture conditions. The transcript abundance of five *PiconPIPs* remained unchanged under control and water-stress conditions in two families that originated from the sites with lower precipitation levels. These two families also displayed a different adaptive strategy of photosynthesis to cope with drought stress, which was manifested by reduced sensitivity in photosynthesis (maintaining the same rate) while exhibiting a reduction in stomatal conductance. In general, root:shoot ratios were not affected by drought stress, but some variation was observed between families. The results showed variability in drought coping mechanisms, including the expression of aquaporin genes and plant biomass allocation among eight families of *Pinus contorta*.

## 1. Introduction

Trees cope with different environmental stresses through diverse physiological and molecular responses. Drought is one of the main abiotic stress factors limiting forest productivity and enhancing insect pests and pathogen-related diseases, increasing forest susceptibility to wildfires and, ultimately, resulting in extensive tree mortality [1,2,3,4,5,6,7,8]. Trees cope with drought stress either through drought avoidance mechanisms that maintain tree water status by preventing water loss or increasing water delivery to leaves, or through tolerance mechanisms which help trees to function under low tissue water potentials when drought levels become too severe [9,10]. Stomatal closure prevents water loss, but also reduces CO_2_ assimilation and negatively affects photosynthesis, ultimately affecting tree growth [11,12,13]. Drought tolerance mechanisms, on the other hand, could lead to tree death if stomata remain open for too long.

Aquaporins (AQPs) are integral membrane proteins that regulate transmembrane water movement in plants [14,15] and play an important role in adjusting the hydraulic conductivity of plant tissues [16,17,18,19,20,21]. These proteins belong to the ancient family of the major intrinsic proteins (MIPs) [14,22], with multiple isoforms present in cell membranes. The plasma membrane intrinsic protein (PIP) and the tonoplast intrinsic protein (TIP) classes are the most abundant aquaporins in plasma membranes and plant tonoplasts [23]. In addition to water, plant aquaporins are also involved in the transport of small molecules with physiological significance across the biological membranes, including silicon (Si), boron (B), O_2_, CO_2_, NH_4_, and H_2_O_2_ [24,25,26]. The ability of aquaporins to transport the small molecules may be crucial in controlling plant responses to biotic and abiotic stress [18].

The functional unit of aquaporins is made up of tetramers under physiological conditions. Each monomer forms a single functional pore made up of six membrane-spanning helices, five connecting loops (loop A–loop E) [27], and two shorter helices HB and HE formed by loops B and E, respectively. The monomer has an hourglass structure made up of an extracellular vestibule, a cytoplasmic vestibule, and an extended pore which connects the two vestibules [28,29]. Most aquaporins contain two highly conserved NPA (Asn-Pro-Ala) motifs [29] and form one of the two constrictions in the channel. Another constriction is formed by aromatic/arginine (ar/R), which is comprised of four amino acid residues. The two constrictions, NPA and ar/R, determine the substrate selectivity [30,31,32]. Spacing between NPA motifs is a selective feature for a substrate, and a precise distance of 108 amino acids (AA) between NPA motifs is essential for silicon transport in plants [33].

Gene expression studies are key for developing a better understanding of plant responses to abiotic stresses. Plants alter gene expression to cope with different environmental stresses [34]. Down-regulation of the expression of specific aquaporins has been shown to be linked to water saving under drought stress [35]. In poplar, drought stress triggered the up-regulation of several aquaporin genes and increased root hydraulic conductance [36]. Changes in aquaporin expression in response to drought stress have also been reported for several other plant species, including strawberry, sugarcane, and common bean [35,37,38]. Several studies reported that various isoforms of plasma membrane intrinsic proteins (PIPs) and tonoplast intrinsic proteins (TIPs) subfamilies were involved in drought stress responses in plants [13,39]. For example, the transcript abundance of *TIP1;1*, *PIP1;2*, *PIP2;1*, and *PIP2;2* varied in response to drought in several plant species [40,41,42]. In this study, we focused on four plasma membrane intrinsic proteins (*PIP1;2*, *PIP2;1*, *PIP2;2*, and *PIP2;3*) and one tonoplast intrinsic protein (*TIP1;1*) in order to better understand the role of these genes in *Pinus contorta* under drought stress.

Although lodgepole pine (*Pinus contorta* Douglas var. *latifolia* (Engelm.)) is one of the most important commercial forest tree species in western North America, its ability to cope with predicted climate change conditions, which includes an increased incidence of drought stress, has not been thoroughly examined. Therefore, a better understanding of drought resistance mechanisms in *P. contorta*, and the subsequent selection of drought-resistant genotypes, is an important strategy for current tree improvement programs and future reforestation efforts. The link between drought stress resistance and *AQP* gene expression has been demonstrated in several plant species. It is important to identify the aquaporins in *P. contorta* that play a role during drought, which could then assist in developing new molecular approaches leading to the selection of broad-spectrum stress-resistant trees.

In this study, we used seeds from eight *P. contorta* mother trees (half-sib families) which are part of a larger tree improvement program in west central Alberta (Western Canada). These parent trees were identified as coming from different site locations spanning the range of environmental conditions based on moisture, for all parents included in the tree improvement program. Based on differences in site origin of the parent trees, we were interested in identifying *PiconAQPs’* responsiveness to drought stress in their progeny under controlled conditions. *PiconAQPs* were cloned from *P. contorta* seedling roots from each family, after 10-weeks of drought and well-watered conditions using qRT-PCR to quantify aquaporin transcriptional responses to drought stress. We also measured physiological traits, growth, and biomass under control (well-watered) and drought stress seedlings of the eight *P. contorta* families. Our results demonstrate a link between aquaporin gene expression and physiological performance in seedlings of *P. contorta* in response to drought stress. We hypothesized that plants originating from sites with a lower summer heat:moisture index (greater water availability), when exposed to drought, would display different adaptive mechanisms to cope with drought stress than plants from sites with a higher summer heat:moisture index (reduced water availability).

## 2. Results

### 2.1. Cloning and Nomenclature

**Figure 1:**
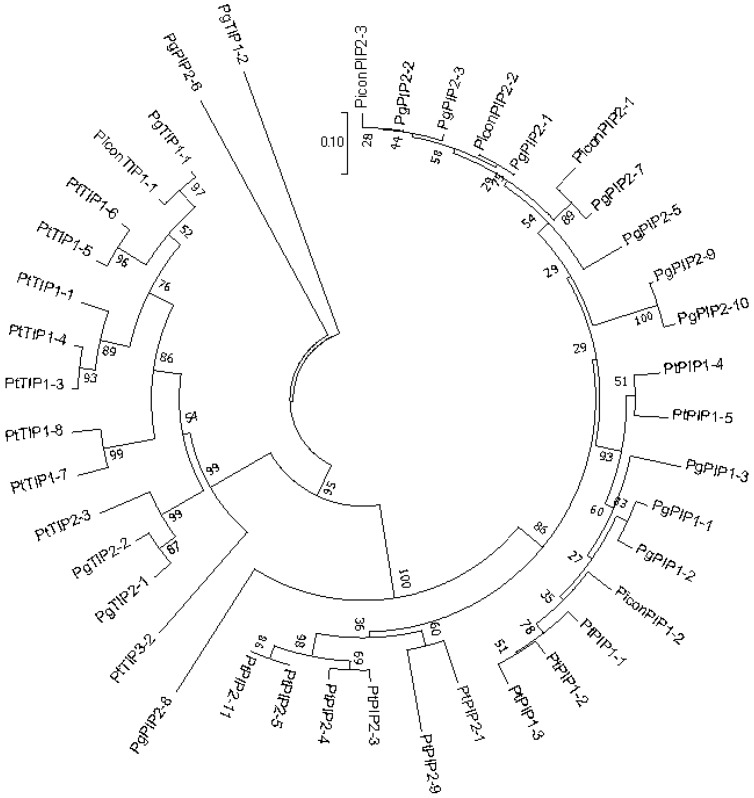
Five *Pinus contorta* (Picon) aquaporins aligned with PIPs and TIPs of *Populus trichocarpa* (Pt) and *Picea glauca* (Pg) amino acid sequences. The unrooted tree was generated with MEGA7.0 [45] using the Neighbor Joining method [46] with 100 bootstrapping replicates. The optimal tree with the sum of branch length = 2.99 is shown.

The root cDNA was used as a template to clone predicted aquaporin genes from *Pinus contorta* using the primers listed in Supplementary Tables S4 and S5. The clones contained open reading frames with a 3-UTR. Five predicted aquaporin genes were cloned from *P. contorta* roots. A BLAST search against the NCBI database revealed that there were three *PIP2*, one *PIP1*, and one *TIP1* aquaporins. The deduced protein sequence of *P. contorta PIP2s*, *PIP1*, and *TIP1* contained 282, 288, and 253 amino acid residues, respectively. The aquaporin genes identified from *P. contorta* were systematically named according to the phylogenetic relationship with the documented aquaporins in *Populus trichocarpa* [43] and *Picea glauca* [44] (Figure 1). Based on the closest *Populus* aquaporin names, *P. contorta* (Picon) aquaporin genes were named as follows: *PiconPIP1;2*, *PiconPIP2;1*, *PiconPIP2;2*, *PiconPIP2;3*, and *PiconTIP1;1*.

### 2.2. Structural Analysis

**Figure 2:**
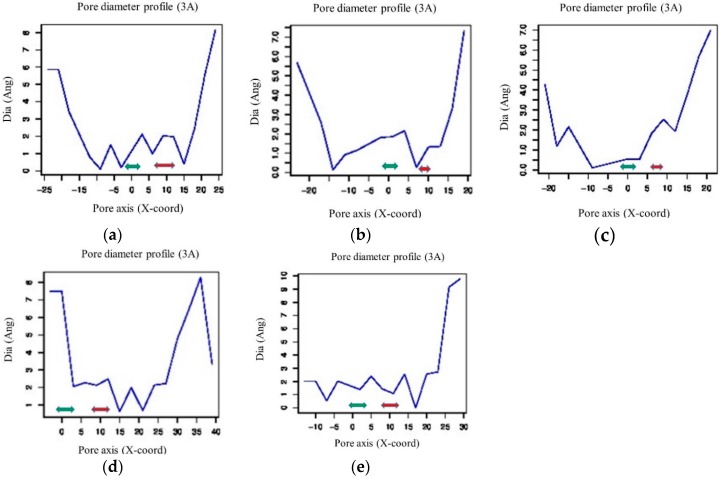

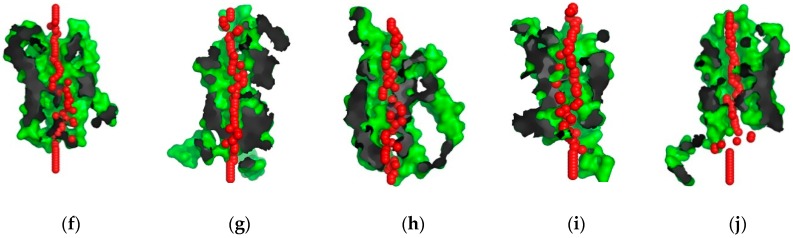
Pore diameter profile (**a**–**e**) of five *Pinus contorta* aquaporins: (**a**) PiconPIP2;1; (**b**) PiconPIP2;2; (**c**) PiconPIP2;3; (**d**) PiconTIP1;1; and (**e**) PiconPIP1;2 at 3A steps obtained from PoreWalker software. The red arrow indicates the approximate location of the ar/R filter and the green arrow corresponds to the NPA sites. The cavity features in the XZ-planar section (**f**–**j**) of five PiconAQPs: (**f**) PiconPIP2,1; (**g**) PiconPIP2;2; (**h**) PiconPIP2;3; (**i**) PiconPIP1;2; and (**j**) PiconTIP1;1 for Y < 0, coordinates only. Red spheres indicate pore centers at 1A step along the pore axis.

Sequence analysis of three PiconPIP2s using the PredictProtein [47] online tool indicated that they all contain six α helices (H1–H6), two half helices (HB and HE), and five loops (LE1–LE5). Functional characterization of the PiconAQPs was conducted by the presence of ar/R, Froger’s residues, and NPA motifs. All five PiconAQPs contained dual asparagine-proline-alanine (NPA) motifs (Figure S1). All PiconPIPs were composed of an ar/R region with phenylalanine in H2, histidine at H5, and threonine and arginine at LE positions (Figure S1; Table 1). In PiconTIP1;1, an ar/R filter was comprised of histidine at H2, isoleucine at H5, and alanine and arginine at the LE position (Figure S1; Table 1). Froger’s positions (P1–P5) in the PiconPIPs were highly conserved glutamine-serine-alanine-phenylalanine-tryptophane (Figure S1; Table 1). Froger’s position in the PiconTIP1;1 was threonine-alanine-alanine-tyrosine-tryptophane (Figure S1; Table 1). The theree-dimensional structure of the pore cavity of five *P. contorta* aquaporins was identified using a computational approach, PoreWalker 1.0 [48] (Figure 2). Figure 2a–e shows the predicted pore diameter of five PiconAQPs at 3A steps. Different pore diameter profiles were obtained for three PiconPIP2s, with substantial variation in pore size and constrictions (Figure 2). The diameter of the pores varied from <1 to 7 Angstroms. PiconAQPs’ pore cavities were visualized using PoreWalker (Figure 2f–j) in the XZ-plane section, with Y > 0 coordinates only. Red spheres indicate pore centres at 1A step along the pore axis.

**Figure 3:**
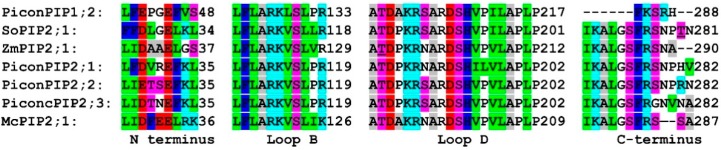
Sequence comparisons of the aquaporins PiconPIP2s, PiconPIP1;2, SoPIP2;1, ZmPIP2;1, and McPIP2;1. The alignment of loops B and D [49]. The first few letters of each AQP name refer to the plant species (Picon, *Pinus contorta*; So, *Spinacia oleracea*; Zm, *Zea mays*; Mc, *Mesembryanthemum crystallinum*). Amino acid residues are colored according to their side chain: large aliphatic (green), small aliphatic (gray), basic (blue), acidic (red), hydroxyl non-aromatic (purple), and aromatic (royal blue). Amino acid residues involved in anchoring of loop D to loop B and the N-terminus are shown. Sequence alignment was done using MUSCLE.

The conserved amino acid residues that were predicted to be involved in water channel regulation in PiconAQPs are shown in Figure 3. A conserved glutamic acid residue was located in PiconPIP2s at Glu32 and in PiconPIP1;2 at Glu45 (corresponds to Glu31 in SoPIP2;1; Glu34 in ZmPIP2;1, and Glu33 inMcPIP2;1). In loop B, a conserved serine residue was located at Ser116 in PiconPIP2s and at Ser130 in PiconPIP1;2 (corresponds to Ser115 in SoPIP2;1; Ser126 in ZmPIP2;1, and Ser123 in McPIP2;1). A conserved histidine residue in loop D was located at His208 in PiconPIP1;2 and at His194 in PiconPIP2s (corresponds to His193 in SoPIP2;1; His204 in ZmPIP2;1, and His201 in McPIP2;1). Identification of these conserved residues will be helpful in revealing the role of PiconAQPs in stress resistance by conducting in vitro functional assays.

### 2.3. Transcript Abundance of Five Pinus Contorta Aquaporins

**Figure 4:**
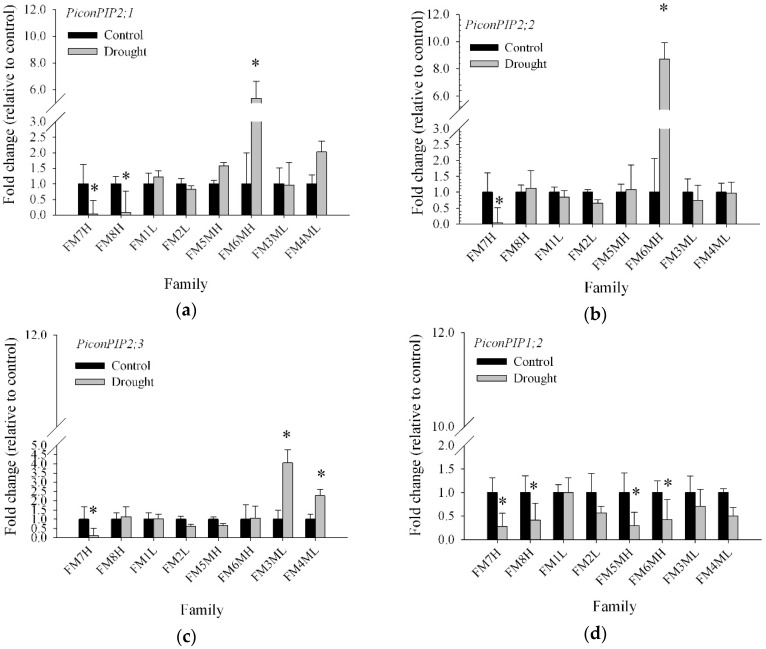

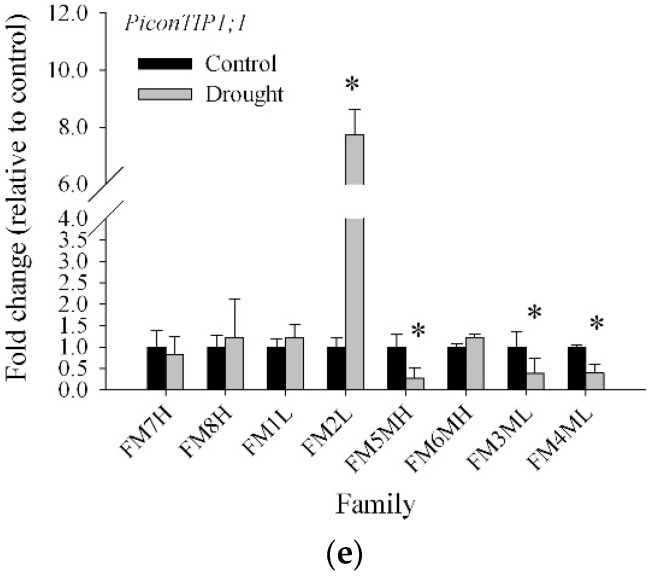
Relative mRNA levels of five aquaporin genes of eight families in *Pinus contorta* roots (expressed as fold change from control) (**a**) *PiconPIP2;1;* (**b**) *PiconPIP2;2;* (**c**) *PiconPIP2;3*; (**d**) *PiconPIP1;2;* and (**e**) *PiconTIP1;1*. Values are for control and drought-stressed plants. Mean values with the asterisks indicate significantly different at *p* ≤ 0.05. Error bars on each column indicate the standard deviation from four biological (seedling) replicates.

In this study, the expression of *PiconAQP* gene transcripts was determined in seedlings from eight *Pinus contorta* half-sib families, including FM1L, FM2L, FM3ML, FM4ML, FM5MH, FM6MH, FM7H, and FM8H (Table 2), subjected to drought stress to shed more light on the possible role of the different aquaporins in their response to drought. The effects of family and drought and of their interaction were determined and are presented in Supplementary Table S2. Transcript abundance of *PiconPIP2;1*, *PiconPIP2;2*, *PiconPIP2;3*, *PiconPIP;1*, and *PiconTIP1;1* was determined by qRT-PCR analyses in root tissue of seedlings in response to drought in the eight families, initially selected based on the summer heat:moisture index of each parent tree location (Table 2). In Figure 4, the fold change of expression between control and drought-stressed plants of all five *P. contorta* aquaporins is shown as mean values from four seedlings per family per treatment after 10-weeks of drought stress. Significant (*p* < 0.001) variation in the transcript abundance of five *PiconAQPs* was observed between the experimental families (Figure 4). Analysis of variance indicated significant interactions (*p* < 0.001) between drought and family in the transcript abundance of *PiconPIP2s* and *PiconTIP1;1* (Table S2). The transcript abundance of these five aquaporins was significantly (*p* < 0.001) different between families under drought stress (Figure 4). Drought had a major impact on transcript abundance of *PiconPIP2;1* and *PiconPIP1;2* (Table S2). Transcript abundance of *PiconPIP1;2* was significantly (*p* < 0.001) lower in drought-stressed plants than in control plants. *PiconPIP1;2* was down-regulated in families FM7H, FM8H, FM5MH, and FM6MH, but was found to be unresponsive to drought in families FM1L, FM2L, FM3ML, and FM4ML. *PiconTIP1;1* was significantly up-regulated, about eight fold, in drought treated family FM2L, whereas *PiconTIP1;1* was significantly down-regulated in families FM5MH, FM3ML, and FM4ML compared to control plants. Transcript abundance of *PiconPIP2;1* and *PiconPIP2;2* appeared to be six and nine fold higher, respectively, in drought treated seedlings in family FM6MH compared to well-watered control plants. On the other hand, *PiconPIP2;1* was significantly down-regulated in families FM7H and FM8H and *PiconPIP2;2* was significantly down-regulated in family FM7H, with no major differences in expression levels observed for the other families.

### 2.4. Net Photosynthesis, Intrinsic Water Use Efficiency, and Stomatal Conductance

**Figure 5:**
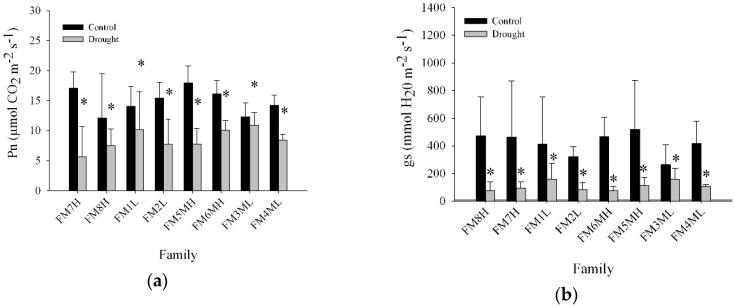

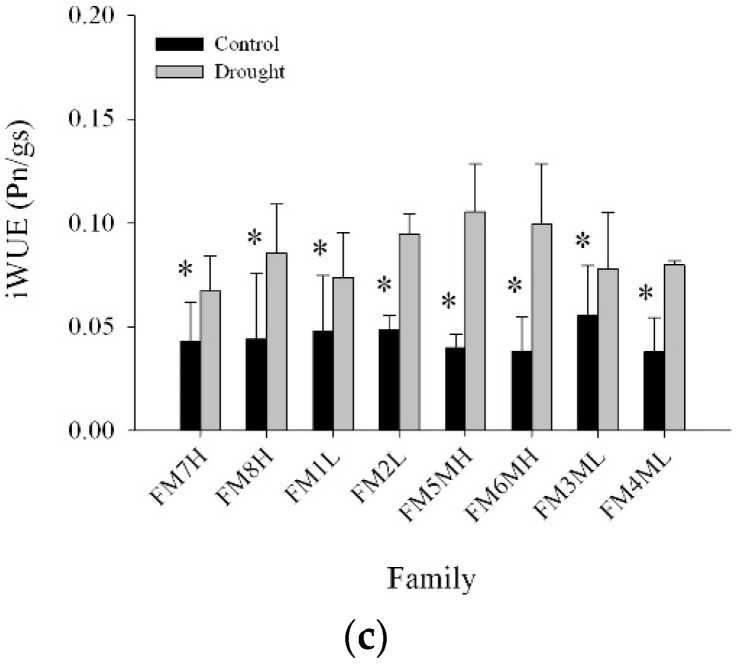
(**a**) Net photosynthesis (Pn), (**b**) stomatal conductance (gs) of needles, and (**c**) intrinsic water use efficiency (iWUE = Pn/gs) from eight *Pinus contorta* families under greenhouse conditions at 63 days after drought treatment initiation (63DAT). Values are means plus the standard deviation with four biological replications. Mean values with the asterisks indicate a significant difference at *p* ≤ 0.05.

**Figure 6:**
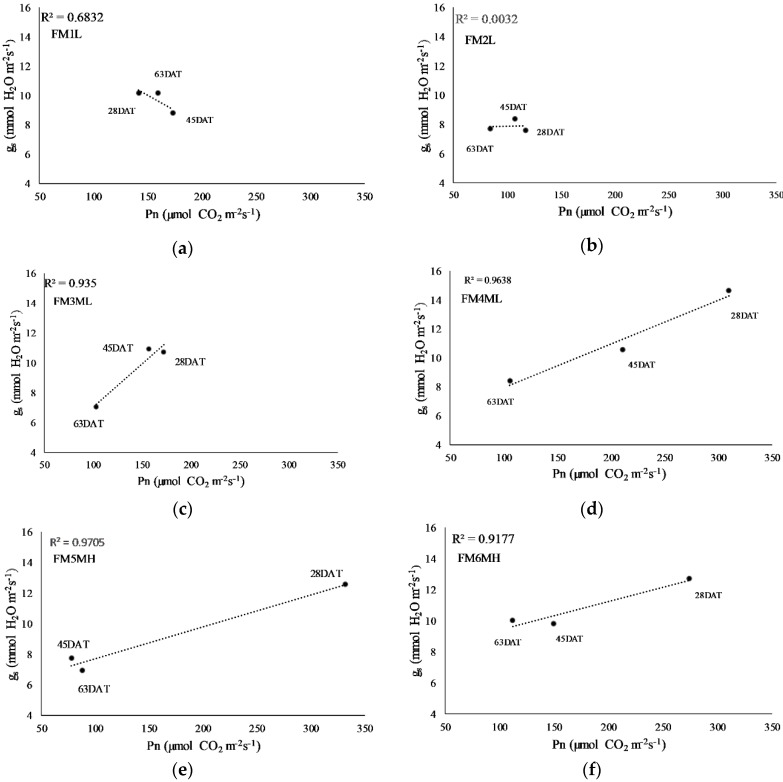

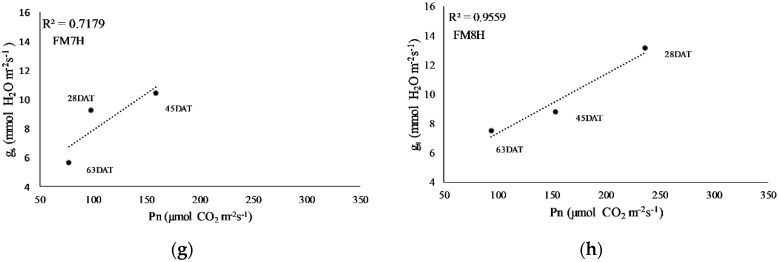
Relation between net photosynthesis (Pn) and stomatal conductance (gs) for eight lodgepole pine families over 63 days of drought stress. The data points represent days after drought treatment initiation (DAT) at 28DAT, 45DAT, and 63DAT: (**a**) FM1L; (**b**) FM2L; (**c**) FM3ML; (**d**) FM4ML; (**e**) FM5MH; (**f**) FM6MH; (**g**) FM7H; and (**h**) FM8H.

Net photosynthesis (Pn) and stomatal conductance (gs) declined significantly compared to controls after 63 days of drought stress in all eight families (Figure 5). All families responded similarly with these parameters and all seedlings responded to drought with an increase in intrinsic water use efficiency (iWUE) and a corresponding reduction in gs (Figure 5). Net photosynthetic rates and gs at 28DAT, 45DAT, and 63DAT were correlated (Figure 6) and exhibited a linear relationship in five of the *P. contorta* families (FM8H, FM3ML, FM4ML, FM5MH, and FM6MH), with the coefficients of determination (r^2^) having values greater than 0.90, whereas a non-linear relationship was found in families FM2L and FM1L (Figure 6).

### 2.5. Growth Parameters and Water Content

**Figure 7:**
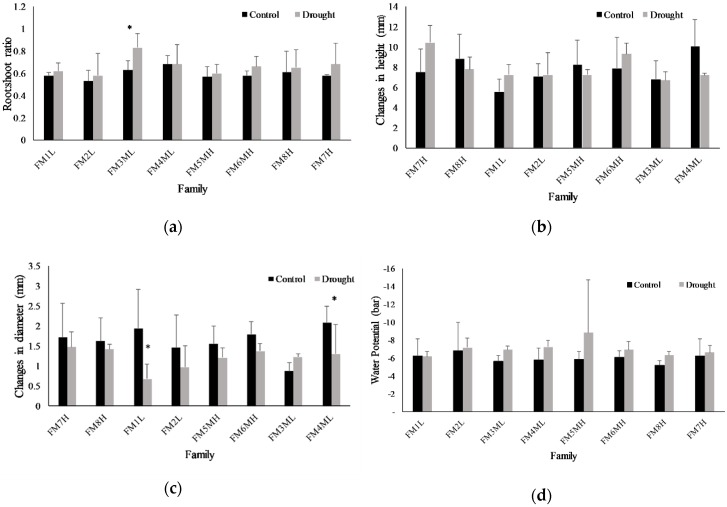
(**a**) Root:shoot ratios of dry biomass, (**b**) changes in height, and (**c**) changes in root collar diameter of eight *Pinus contorta* families exposed to two watering treatments: well-watered controls and drought. Values are means plus the standard deviation with four biological replications. Mean values with the asterisks indicate significant difference at *p* ≤ 0.05. (**d**) Plant water potential (bars + SD; n = 4) at 63DAT just prior to harvesting for control and drought treated *Pinus contorta* seedlings.

Root:shoot ratios showed no significant differences between treatments after harvest at 63DAT, although drought-treated plants tended to show larger root:shoot ratios compared with controls (Figure 7). On the other hand, a significant family effect was observed for the root:shoot ratios (Table S1). Seedling height and root collar stem diameter were recorded at 1DAT and at 63DAT for all plants. The results were reported as changes in height and diameter between day 1 and 63 (Figure 7). There were no significant differences in seedling height or root collar diameter between control and drought-stressed seedlings (Table S1) and the family effect was observed for changes in height only (Table S1).

## 3. Discussion

Aquaporins are integral membrane proteins which facilitate the permeation of water and some other small molecules [25,39]. The structural features of aquaporins have been established using the resolved aquaporin crystal structures from plant [31], mammalian [50,51,52], bacteria [53,54], yeast [55], and archaeal [56] organisms. Until now, the crystal structures of two plant aquaporins, one from *Spinacia oleracea*, SoPIP2;1 [31], and another from *Arabidopsis thaliana*, AtTIP2;1 [57], have been resolved. The lack of advancement in the discovery of additional aquaporins in plants may be because X-ray crystallography, needed to elucidate these structures, is a time consuming technique that requires extensive expertise. The recent development of computational tools has made it possible to by-pass X-ray crystallography, allowing for the prediction of protein structures and structural features using in-silico approaches. The three-dimensional (3D) structural fold of a protein family is evolutionarily conserved [58]. Therefore, it is relevant to analyze the structure of a protein to gain insight into its biological function. Previous studies showed that PIPs and TIPs were involved in regulating root water transport, hydraulic conductivity, and plant water relations and are also responsive to salt and drought stress [14,39]. Several studies showed that plants overexpressing certain PIPs and TIPs increased their drought resistance by decreasing their transpiration rates and stomatal conductance [59,60,61]. Contradicting results have been reported for the effects of aquaporin overexpression under abiotic stresses, with either positive or negative effects on plant stress resistance [14,62,63,64]. In this study, five aquaporins, including *PiconPIP2;1*, *PiconPIP2;2*, *PiconPIP2;3*, *PiconPIP1;2*, and *PiconTIP1;1* from *Pinus contorta*, were cloned and analysed in-silico to gain insight into their biological function. Sequence analysis showed that PiconAQPs possess two NPA motifs (Figure S1) which form the central constriction of the pore that allows water-selective permeation in AQP water channels and excludes protons and cations [65,66]. The ar/R region of all four PiconPIPs showed very high similarity to other plant species, including Arabidopsis and flax [67,68], with phenylalanine at H2, histidine at H5, and threonine and arginine at loop LE (Figure S1). The ar/R filter composed of phenylalanine, histidine, threonine, and arginine is characteristic of water-selective aquaporins that are unable to transport other small solutes [49,69,70]. *Mesembryanthemum crystallinum*, McPIP2;1 with the aromatic/arginine (ar/R) region with phenylalanine, histidine, threonine, and arginine showed a 14-fold increase in water permeability across the oocyte plasma membrane, but was impermeable to glycerol and urea [49]. In barley, five aquaporins, PIP2s, HvPIP;2, HvPIP2;1, HvPIP2;2, HvPIP2;3, and HvPIP2;5, were found to be permeable to water and CO_2_ when expressed in *Xnopus laevis* oocytes [71]. TIP subfamilies showed a diverse composition of residues within the ar/R regions. In Arabidopsis, TIPs were subdivided into three groups based on the residues of the ar/R filter [68]. The amino acid residues composition of PiconTIP1;1 was similar to Arabidopsis TIPs in Group IIB. In the flax TIP sub-family, the H2 position of the ar/R filter was comprised of histidine and the H5 position was comprised of isoleucine. PiconTIP1;1 showed similarity to LuTIP3s, which contained an ar/R filter with histidine, isoleucine, alanine, arginine residues and less hydrophobic ar/R compared to LuTIPs and LuTIP1s, with the residues histidine, isoleucine, alanine, valine and histidine, isoleucine, alanine, and leucine, respectively [67]. The polarity of the H2 and H5 positions of the ar/R of TIPs is reversed from PIPs [68]. Five Froger’s positions (Figure S1; Table 1) showed that the PiconPIPs likely perform similar biological functions. Pore-forming conserved amino acids determine the substrate specificity because these residues are responsible for pore diameter and pore hydrophobicity [31,72]. PiconTIP1;1 has two constrains similar to LuTIP3s that are known to act as a selectivity barrier in the pore [67]. However, the size of the pore and the position of the constrain were different in PiconTIP1;1 from LuTIP3s. The cavity diameter of PiconTIP1;1 was about 7A, which is about three times the size of a water molecule (Figure 2). Arabidopsis TIP3-2 and TIP3-1 are suggested to have dual functionality on water and H_2_O_2_ permeability, but their physiological roles are still unknown [73]. In all PiconPIPs, the diameter of the pores varied from <1 to 7A (Figure 2), which refers to the regulated movement of water through the channel. Moreover, different pore diameter profiles were obtained between three PiconPIP2s (Figure 2). These results indicated that the three PiconPIP2s might be governed by different molecular mechanisms. For example, the transcript abundance of *PiconPIP2;3* was upregulated in families FM3ML and FM4ML, whereas the transcript abundance of *PiconPIP2;1* and *PiconPIP2;2* remained unchanged. Plant aquaporins possess gating mechanisms that maintain the water movement in plants by turning it on and off. The gating of water channels (aquaporins) is regulated by the phosphorylation of serine residues and protonation of histidine residues in loop D. Conserved serine residues were located at Ser116 in PiconPIP2s and at Ser130 in PiconPIP1;2. It was reported that the Ser123 (corresponding to Ser116 in PiconPIP2s and Ser130 in PiconPIP1;2) was essential for the water channel activity in McPIP2;1 [49]. Changes in cytoplasmic pH causes protonation of the conserved histidine residue in loop D that leads to the closure of aquaporin channels. The conserved histidine residue was located at His208 and His194 in PiconPIP1;2 and PiconPIP2s, respectively (Figure 3). It will be essential to understand the functional properties of these aquaporins before a full understanding of their significance in drought resistance processes can be determined. Moreover, different aquaporin isoforms contribute differently to water transport and regulation. It is also noteworthy that PiconPIP2;1, PiconPIP2;2, and PiconPIP2;3 have different pore structures when compared with each other (Figure 2).

Transcript profiling of aquaporin genes could provide valuable insights into the molecular basis of phenotypic differences in plants in response to drought. In this study, the first report on aquaporin expression patterns in a genetically diverse population of plants is provided. To monitor possible differences in the transcript abundance of five *PiconAQPs* due to drought stress, eight *P. contorta* families were chosen based on the summer heat:moisture index of the parent origin location (Table 2). Diverse transcript abundance patterns were observed among the five *PiconAQPs* because some aquaporins were up-regulated, which can help plants maintain normal physiological processes by avoiding drought stress, while other aquaporin isoforms are down-regulated so that plants can tolerate dehydration during drought stress [13,74]. The expression levels of *PiconPIP2;1, PiconPIP2;2*, and *PiconPIP2;3* were significantly down-regulated in family FM7H. The decrease in the membrane abundance of these aquaporins could be expected to decrease the water permeability of the plasma membrane, thereby decreasing the water loss and maintaining turgor during drought stress. On the other hand, *PiconPIP2;1*, and *PiconPIP2;2* were significantly up-regulated in family FM6MH and *PiconPIP2;3* was significantly up-regulated in families FM3ML and FM4ML. Up-regulation of *PiconPIP2s* in the seedlings of these families suggests that *PiconPIP* aquaporins may enhance water uptake to maintain the plant water status. Interestingly, the expression levels of all four *PiconPIPs* remained unchanged in families FM2L and FM1L (Figure 4). These two families originated from parents located in the areas where the severity of drought based on the SH:M index was lower than for the other families. The transcript abundance of *PiconPIP1;2* was down-regulated in all seedlings originating from parents located in mid to severe drought areas, whereas other seedlings remained unresponsive to drought in families with parents that originated from areas with less severe drought stress (Figure 4; Table 2). In several studies, *PIP1* isoforms were reported to be associated with CO_2_ and O_2_ transport [26,75,76]. Transcript levels of the tonoplast aquaporin, *PiconTIP1;1*, was down-regulated by drought in families FM3ML and FM4ML and significantly up-regulated in family FM2L. It was noticeable that the SH:M index of family FM2L was 29.6, whereas for families FM3ML and FM4ML, it was 32.1 and 32.2, respectively (Table 2). This result suggests that local adaptation may lead to variation in gene expression levels within the population of *P. contorta* studied. Transcript profiling of five *PiconAQPs* provided strong evidence of differential expression patterns among the studied *P. contorta* families. The underlying causes for the *PiconAQP* expression variations within the population could be very complex. Several studies have shown that epigenetic mechanisms could help plants to adapt to different environments and the evolution of phenotypic plasticity could also be playing a key role [77,78]. Differential gene expression was reported in response to salt stress among and within *Phragmites australis* clones that could also be due to epigenetic variation [79]. Based on the present study, the potential role of epigenetics within the population is certainly worthy of further exploring. The exact contributions of five Picon aquaporin genes in drought stress coping mechanisms are still illusive. Numerous reports showed that the correlation between mRNA and protein abundance is poor. As a result, mRNA expression may not accurately measure protein-function levels [80,81,82,83]. Moreover, lipid composition and membrane fluidity changes could modify the functionality of aquaporins [84,85].

In terms of gas exchange and seedling water status, net photosynthesis and gs (Figure 5) decreased with prolonged drought stress, indicating that all the *P. contorta* families in this study decreased water loss and delayed the decline in their water potential in response to drought stress. As a result, seedling water potential was not significantly decreased after 63 days of drought stress compared to controls (Figure 5), helping seedlings to maintain water potential and increase their resistance to drought stress. These results showed common plastic responses to water stress, suggesting a conserved plastic strategy at the population level. However, divergent patterns at the family level were identified beyond the former plastic responses. For family FM6MH, gs decreased and root *PiconPIP2;1* and *PiconPIP2;2* expression levels increased in drought treated plants (Figure 4 and Figure 5), indicating increased water uptake and decreased water loss. On the other hand, for family FM7H, gs decreased, while root *PiconPIP2;1* and *PiconPIP2;2* expression levels decreased as well. These results suggest genetic variability in the mechanisms to cope with drought in the studied population of *P. contorta* from a restricted region on the eastern slopes of the Canadian Rockies. The correlation between Pn and gs of these two families (Figure 6) also supports this finding. A positive correlation was obtained for families FM7H and FM8H, whereas no correlation was observed between Pn and gs for families FM1L and FM2L. There was no significant difference in changes in height between the control treatment and drought-stressed trees, whereas diameter growth was significantly lower at the 5% level (Table S1). Plants commonly respond to drought stress by increasing their root:shoot ratios [61,86,87], which is an important parameter to assess the stress avoidance capacity of plants. Interestingly, our findings showed that the applied drought treatment in our study did not lead to a decrease in total biomass of *P. contorta* seedlings or shift in the root:shoot ratio. This suggests that the drought treatment did not reach the threshold limit required to change the biomass carbon allocation. However, inconsistent results with the optimal partitioning theory were also found in other work with drought-adapted forest tree species [88]. This observed pattern essentially represents a drought avoidance strategy that is characterized by stomatal closure before a significant decrease of leaf water potential [89], slowing down growth and adjusting plant size in response to the reduced amounts of assimilated carbon [88,90]. On the other hand, a significant family effect was observed for the root:shoot ratio (Table S1), showing strong within-population genetic variation in seedling carbon allocation patterns. These findings demonstrate again that different strategies coexist within the studied population of *P. contorta* to cope with drought stress.

Transcript profiling of *PiconAQPs*, gas exchange regulation and carbon allocation provided strong evidence of differential patterns among the *P. contorta* families coming from a reduced area of the distribution range of the species. These results show within-population genetic variation in the transcription of proteins and phenotypic responses involved in drought stress coping mechanisms. Thus, it suggests coexistence of divergent strategies to cope with drought stress, showing the complexity of such mechanisms within tree populations. Actually, trees show high intrapopulation genetic diversity relative to other plant species [91] and such diversity is critical to buffer changes in selective pressures due to environmental variation over the extended lifespan of trees [92] and likely contributes to their evolutionary success [93]. A number of mechanisms have been proposed for the maintenance of genetic variation of traits under selection [94]. These include pleiotropy [95], genetic correlations with multiple traits [96], trade-offs during ontogeny [97], temporal variation in selection [98], and environmental heterogeneity [99]. Ultimately, high within-population genetic diversity in long-lived species, including trees, is critical to buffer stochastic selective events such as severe droughts, the frequency and intensity of which are expected to increase with climate change [100]. Understanding the function and effects of the studied *AQPs* on drought resistance in tree species is of paramount importance for tree breeding programs. Taking advantage of *AQPs* expressions as a proxy of tree resistance to drought would allow selection for increased resistance, permit the maintenance of genetic diversity in drought resistance mechanisms, and support the adaptive potential of planted forests subjected to a variety of current and future threats.

## 4. Materials and Methods

### 4.1. Plant Material and Growth Conditions

**Figure 8:**
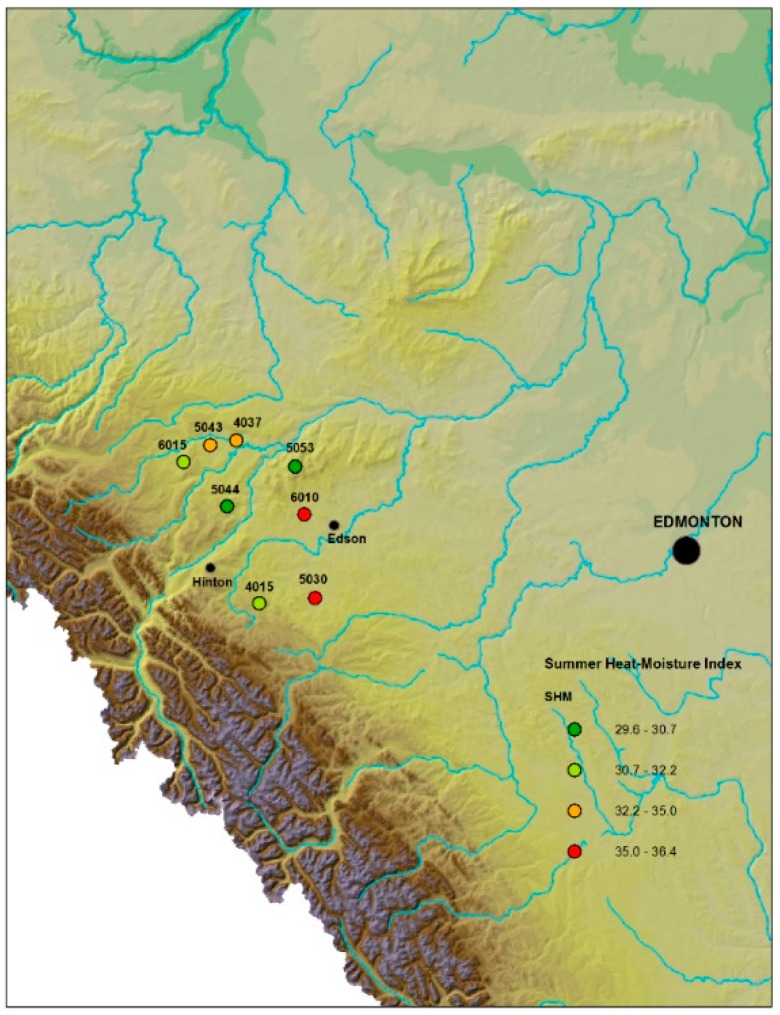
Source sites of *Pinus contorta* families from the eastern slopes of central Alberta Rocky Mountains (Canada; Region A *Pinus contorta* Controlled Parentage (tree improvement) Program (CPP) used in this study. ArcGIS 10.5 GIS was used to create the site map. The map shows the unique identifier number of *P. contorta* families and summer heat:moisture index (SH:M) of the region.

A population of *Pinus contorta* Douglas *var. latifolia* (Engelm.) seeds were collected from eight open-pollinated, wild mother trees from eight different locations on the eastern slopes of central Alberta Rocky Mountains (Canada; Region A *Pinus contorta* Controlled Parentage (tree improvement) Program (CPP); see Figure 8) following a water availability gradient that matched an elevation gradient in the region. Mother trees were selected in the wild based on phenotypically superior growth and form and were assumed to be unrelated, thus seeds from each individual mother tree were considered half-siblings with an unknown pollen parent source. The eight families were classified based on a drought severity index from the original location of their mother, determined using a summer heat:moisture index (SH:M). This index was calculated as follows: SH:M = ((MWMT)/(MSP/1000)), where SH:M is the summer heat:moisture index, MWMT is the mean warmest month temperature (°C), and MSP is the mean summer (May to September) precipitation (mm). Families were classified accordingly as (Table 2): Low severity (SH:M =< 31); Low-mid severity (SH:M = 31–33); Mid-high severity (SH:M = 33–35); and High severity (SH:M => 35). Table 2 shows the unique identifier number and geographical origin of the families. For the convenience of data presentation, we have renamed the families as follows: FM1L, FM2L, FM3ML, FM4ML, FM5MH, FM6MH, FM7H, and FM8H (Table 2). Here, FM: family; 1–8: number of family; and L, ML, MH, and H: Low severity, Mid-low severity, Mid-high severity, and High severity, respectively.

Seeds were soaked for 24 h in a water bath at 22 °C and stored at 4 °C for two weeks to promote germination. Seeding occurred in early May 2016 in a commercial forest nursery (Bonnyville, AB, Canada; 54,275° N 110,756° W; 550 m a.s.l.) and grown in 410A styro-blocks. After seven months of growth, in late November 2016, seedlings were lifted, bundled, and moved to cold storage at −3 °C for two months until being transported to a greenhouse located at the University of Alberta (Edmonton, AB, Canada; 53,527° N 113,529° W; 670 m a.s.l.), where the study was conducted. Seedlings were potted up into 2,000 cm^3^ pots filled with 2.700 g of fine play sand (quikrete premium play sand, QUIKRETE^®^), with a mesh cloth in the bottom to avoid sand and root loss. Six grams of slow release fertilizer (Multicote^®^ 8, NPK 17:7:14 + minors, Haifa^®^) was spread over the surface of every pot. To maintain the nutrient balance throughout the experiment and lower the pH of the substrate, seedlings were watered with pH reducing fertilizer (Plant-Prod Solutions 18-9-18 pH Reducer) at a rate of 50 mg kg^−1^ twice during the experiment. Seedlings were grown for two months prior to the initiation of drought treatments under natural light supplemented with a photosynthetic flux density of 400 μmol m^−2^ s^−1^ at the top of the plants to maintain a 16 h light:8 h dark photoperiod [88]. The relative humidity ranged from 42 to 98 % (daily average 82%). During the two-month period, control seedlings were watered every other day.

### 4.2. Drought Treatments and Experimental Design

**Figure 9:**
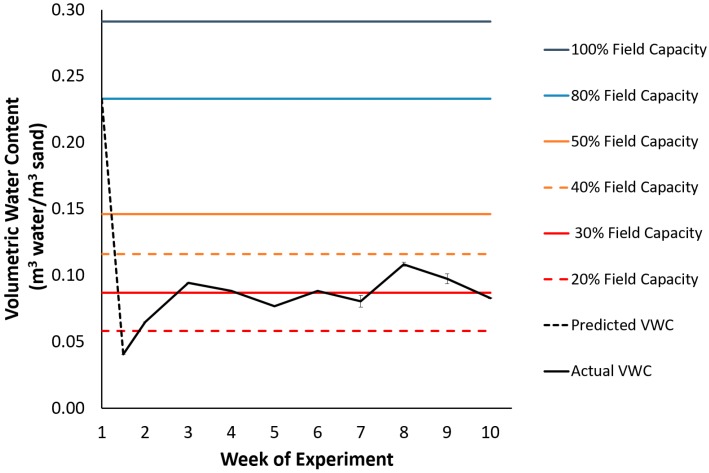
Volumetric water content (m^3^ water/ m^3^ sand) of the drought treatment during the period of drought treatment in the experiment, as measured with a theta moisture probe.

Seedlings were arranged in a split-plot design replicated in two blocks with two water availability treatments (whole plot) and eight half-sibling families as the split factor (split-plots). In every block, two seedlings per family were randomized for a total of 64 seedlings. Half of the plants were subjected to a stress-free watering regime (control), while the other half were subjected to a water deficit treatment. In the control treatment, the substrate was kept at field capacity throughout the whole experiment, while in the drought-stress treatment, the substrate was kept at 30–40% of field capacity during the experimental period of drought (Figure 9). The amount of water supplied in the drought treatment was determined first by measuring the volumetric water content (VWC) of pots from each drought treatment pot with a moisture probe (ML3 ThetaProbe Soil Moisture Sensor, Delta-T Devices) to calculate a mean VWC per plot. Then, the mean water volume in the pots was calculated based on the mean VWC. Finally, the difference in water volume required to return the substrate back to the desired moisture level was calculated and rounded to the nearest 5 mL.

### 4.3. Measurements

Gas exchange measurements were performed using an infrared gas analyzer (IRGA; Ciras-3, PP Systems) by employing the conifer cuvette on a subset of trees (one seedling per family per block-treatment combination) during each of the three measurement periods: 28DAT, 45DAT, and 63DAT. Two fascicles (four needles) per seedling were randomly selected (one old fascicle selected from the lower portion of the seedling and one new fascicle selected from the current year needles towards the top of the seedling). Net photosynthesis (Pn) was expressed as a function of needle surface area [101]. Needle area was calculated for each sample using a scanner (Epson Perfection V800 Photo Scanner) and WinSEEDLE software (Regent Instruments Inc., Quebec, QC, Canada). From gas exchange and leaf area measurements, net photosynthesis (Pn), stomatal conductance (gs), and intrinsic water use efficiency (iWUE) were calculated (iWUE = Pn/gs).

Water potential was measured at the time of harvest (63DAT) collection for *PiconAQPs* expression analysis using the method described earlier [102]. In brief, stems were cut about 2.5 cm above the soil surface, stripped of their bark and shoots, and inserted into the pressure chamber (Scholander-type pressure chamber Model 600 PMS instruments, Corvallis, OR, USA), and dry air was used for applying pressure. Stems were visually observed with a magnifying glass, and measurements were taken when water droplets formed at the cut surface after approximately 5 to 10 s.

Roots were washed, sieved, thoroughly patted dry, and allowed to air dry for approximately four hours before weighing. Needles were removed carefully to keep the fascicle on the stem intact. Biomass was oven dried at 60 °C for 24 h before measuring dry weights of roots, shoots, and needles. Root:shoot ratios were calculated as the ratio of dry root biomass to dry needle and shoot biomass combined.

### 4.4. Database Sources

Predicted *Pinus contorta* AQP gene sequences were obtained from the available expressed sequence tags of *P. contorta* in the Expressed Sequence Tags (ESTs) database at the National Center for Biotechnology Information (NCBI) and by a blast search of Arabidopsis aquaporins against the *Pinus taeda* genome, a close relative of *Pinus contorta*, at Congenie.org. Tertiary protein structure predictions were conducted by submitting the protein sequences of PiconPIP2;1, PiconPIP2;2, PiconPIP2;3, PiconPIP1;2, and PiconTIP1;1 to the I-TASSER Standalone Package [103]. The results obtained in the Protein Data Bank (PDB) format from I-TASSER were uploaded to the PoreWalker server [48] to predict pore shape and cavity structure. Conserved sequences for substrate specificity of Froger’s positions (P1–P5), NPA motifs, ar/Rfilters (H2, H5, LE1, LE2) were identified by manual examination of multiple sequence alignments of PiconAQPs with structurally characterized AQPs, as reported earlier [67,104,105].

### 4.5. RNA Extraction, cDNA Synthesis, and RT-qPCR Analysis

A total of 64 root tissue samples were collected from eight families (Table 2), two treatments (well-watered and drought), and four seedlings per family, at the end of the experiment (63DAT). Roots were washed carefully using distilled water to remove any sand and blot-dried with a paper towel. The collected tissues were flash-frozen immediately in liquid nitrogen. The root samples were collected within a two-hour time period from 9:00 am to 11 am to minimize potential transcriptional variation due to diurnal effects. The root tissue samples were collected approximately 12 cm from the root tip. After removing from liquid nitrogen, tissue samples were ground with a mortar and pestle in liquid nitrogen. Total RNA was extracted from roots using a combination of cetyltrimethylammonium bromide (CTAB) and the QIAGEN RNeasy Plant Mini Kit (Qiagen, Valencia, CA, USA) [106]. RNA concentration and purity were assessed using the Thermo Scientific™ NanoDrop™ One Microvolume UV-Vis Spectrophotometer (Thermo Scientific, Waltham, MA, USA). RNA quality was also checked on a 1% (*w*/*v*) agarose gel. The removal of genomic DNA contamination and first strand cDNAs were generated using the QuantiTect Reverse Transcription Kit (Qiagen, Valencia, CA, USA) with 500 µg total RNA, according to the manufacturer instructions. The qRT-PCR was performed using SYBR Green I dye reagent in an Applied Biosystems 7500 Fast system with 10-fold diluted cDNA. The PCR conditions were as follows: 1 cycle at 95 °C for 2 min, followed by 40 cycles at 95 °C for 0.15 s and at 60 °C for 1 min. The relative expression of all genes was calculated using the delta-delta-Ct method (2^−ΔΔCT^). Two reference genes were selected that are commonly used for normalization in real-time PCR applications in other plant species. These include elongation factor 1-alpha and actin. The specific primers were designed using the PrimerQuest tool of the Integrated DNA Technologies. Four biological replicates (seedlings per family and treatment combination), each with three technical replicates, were analysed. Primers used for qRT-PCR are listed in Supplementary Table S3. Melting curve analysis of the reaction was performed in order to verify the single PCR product amplified for each set of primers. To further confirm the specificity of the primer sets, the qRT-PCR products were separated and visualized by 2% agarose gel electrophoresis. Standard curves were used to calculate the gene-specific PCR efficiency from 10-fold series dilution for each primer pair. The linear regression correlation coefficient (r^2^) and slope values were calculated from the standard curve, and PCR amplification efficiencies (E) were calculated according to the equation E = (10 − 1/slope − 1) × 100%.

### 4.6. Rapid Amplification of cDNA 3′ End and Cloning

The first-strand cDNAs were synthesized from 1 μg of total RNA using Superscript II reverse transcriptase (Invitrogen) and an oligo (dT) primer. Coding sequences of putative *PiconAQPs* were amplified with Phusion DNA Polymerase using the primers listed in Supplementary Table S4. The PCR products of an expected size were eluted from the gel and purified using The Wizard^®^ SV Gel and PCR Clean-Up System (Promega, Madison, WI, USA). The PCR products were then cloned into a pCR2.1-TOPO vector using the Topo TA Cloning kit (Invitrogen, Carlsbad, CA, USA) and transformed into DH5α chemically competent cells (Invitrogen, Carlsbad, CA, USA). About three to six white colonies were sequenced for each PCR product using M13 sequencing primers. The coding sequences of PiconAQPs were extended by 3′ rapid amplification of cDNA ends (RACE) [107]. Primers used for RACE are listed in Supplementary Table S5. The nucleotide sequence and translation of clones have been submitted to the GenBank database (accession no. MG938584-MG938588).

### 4.7. Nomenclature of Pinus contorta Aquaporin Genes

For the classification of the identified putative *Pinus contorta* aquaporins, phylogenetic analyses were performed with the known *Populus trichocarpa* and *Picea glauca* AQP protein sequences. Amino acid sequences were aligned with five PiconAQPs identified in this study using the ClustalW program. A tree was constructed using the neighbor-joining (NJ) method in the MEGA7 software suite (http://www.megasoftware.net/mega.html).

### 4.8. Statistical Analyses

The results of the experimental parameters are expressed as means plus standard deviations of biological replicates. To test the significance of watering treatments and family effects, a repeated measures analysis of variance (ANOVA) was carried out using PROC MIXED. The mixed model was fitted with the MIXED procedure of SAS and was performed using SAS Version 9.1 software (SAS Institute, Cary, NC, USA). The main factors watering treatment (drought) and family were considered fixed effects, whereas blocks were considered as a random effect in order to consider the appropriate error terms for each fixed factor. The results were plotted using SigmaPlot 11.0 (Systat Software Inc., Chicago, IL, USA) and Microsoft Excel.

## 5. Conclusions

The expression analyses of several *AQP* genes in eight different *Pinus contorta* families were conducted to decode their underlying molecular mechanisms in response to drought. Eight families of *P. contorta* were selected from locations with different climactic conditions based on the summer heat:moisture index. Five aquaporin genes were cloned, including four PIPs and one TIP. The amino acid composition of the ar/R filter of all four PiconPIPs indicated that these proteins play a role in water transport activity. Ser116 in PiconPIP2s and Ser130 in PiconPIP1;2 may be required for PiconPIP water permeability. The pore cavity diameter varies throughout the pore, indicating that the water transport mechanism is regulated. Differential expressions of *PiconPIP2;1*, *PiconPIP2;2*, *PiconPIP2;3*, *PiconPIP1;2*, and *PiconTIP1;1* were observed among the families in response to the applied drought stress. Moreover, the data from the root:shoot ratio and the root diameter indicates variation in the carbon allocation among the families under varying water deficit conditions. Together, these results suggest that changes in the physiological responses and several aquaporin gene expression patterns are important strategies to cope with drought stress within the population of *P. contorta*. The applied drought treatment was not sufficiently severe to elicit differences in responses or the seedlings did not undergo the stress long enough to show a growth response in height, although they did show a difference in root collar stem diameter. As all trees were harvested at the end of the experiment, it is not possible to know if a response in reduced height growth would have manifested itself in the new growth the following year. It is also clear that *P. contorta* growing in the foothills of the Rocky Mountains, and originating from a variety of site origins and conditions, has the capacity to withstand significant drought at the crucial seedling stage of development.

Obviously, many aspects concerning the molecular and biological function of *PiconPIP2;1*, *PiconPIP2;2*, *PiconPIP2;3*, *PiconPIP1;2*, and *PiconTIP1;1* need to be clarified and require experimental evidence. To further investigate the role of *PiconAQPs*, it would be important to conduct functional assays of these genes using heterologous systems. Moreover, epigenetic analysis in combination with transcript profiling of several other *PiconAQPs* could be a strategy to identify adaptation mechanisms of *P. contorta* families under drought.

## Figures and Tables

**Table 1:** ar/R selectivity filters and Froger’s residues of five *Pinus contorta* aquaporins.

Aquaporin	ar/R Selectivity Filter				Froger’s Positions (P1–P5)				
	H2	H5	Loop E1	Loop E2	P1	P2	P3	P4	P5
PiconPIP2;1	F	H	T	R	Q	S	A	F	W
PiconPIP2;2	F	H	T	R	Q	S	A	F	W
PiconPIP2;3	F	H	T	R	Q	S	A	F	W
PiconPIP1;2	F	H	T	R	Q	S	A	F	W
PiconTIP1;1	H	I	A	R	T	A	A	Y	W

**Table 2:** Families (n = 8) with unique identifiers (UI) were selected from a range of locations (latitude, longitude, elevation) and climate based on the summer heat:moisture index (SH:M) of the region. The present study classified the drought severity (low, low-mid, mid-high, high) based on the SH:M and assigned names accordingly to the families. The details are provided in methods Section 4.1.

UI	Latitude °N	Longitude °W	Elevation (m)	SH:M	Drought Severity	Assigned Name
5044	53.690000	−117.364	1317	30.7	Low	FM1L
5053	53.901944	−116.794	1330	29.6	Low	FM2L
4015	53.208333	−117.07	1313	32.1	Low-mid	FM3ML
6015	53.907500	−117.749	1196	32.2	Low-mid	FM4ML
6010	53.661667	−116.709	1000	35.4	High	FM7H
5030	53.241389	−116.603	1036	36.4	High	FM8H
4037	54.025278	−117.305	1026	35.0	Mid-high	FM5MH
5043	53.996389	−117.525	1047	34.2	Mid-high	FM6MH

## Supplementary Materials

Supplementary materials can be found at http://www.mdpi.com/2223-7747/8/1/13/s1.

## Abbreviations

PIP	plasma membrane intrinsic protein
TIP	tonoplast intrinsic protein
PiconAQPs	*Pinus contorta* aquaporins
DAT	days after treatment
Picon	*Pinus contorta*
AQPs	Aquaporins
ar/R	aromatic/arginine
