# Supplementary material for: Variation in Aquaporin and Physiological Responses Among Pinus contorta Families Under Different Moisture Conditions
Source: Plants (Basel). 2019 Jan 6;8(1):13. doi: 10.3390/plants8010013 (PMC6359517; doi:10.3390/plants8010013)
Supplement: Supplementary file 1 [file plants-08-00013-s001.pdf]

Table S1. Results of a two-way ANOVA (family, drought and family x drought) for root:shoot, stomatal conductance, net photosynthesis, water use efficiency, and changes in height and diameter of eight *Pinus contorta* families displaying source of variation, F-value and *p*-value. Significant sources of variation are highlighted in bold.

Source of Variation	F-value	*p*-value
**Root:Shoot**		
Family	2.520	**0.023**
Drought	0.760	0.388
Family × Drought	0.550	0.788
**Stomatal conductance (gs)**		
Family	0.490	0.823
Drought	39.180	**<0.001**
Family × Drought	1.360	0.314
**Net photosynthesis (Pn)**		
Family	0.820	0.569
Drought	22.120	**<0.001**
Family × Drought	0.780	0.605
**Water use efficiency (iWUE)**		
Family	0.660	0.703
Drought	70.320	**<0.001**
Family × Drought	1.360	0.226
**Changes in height**		
Family	2.220	0.049
Drought	0.080	0.775
Family × Drought	2.030	0.070
**Changes in diameter**		
Family	1.49	0.194
Drought	9.73	**0.003**
Family × Drought	1.30	0.269

Table S2. Results of two-way ANOVA (family, drought and family x drought) for relative gene expression of five aquaporin genes (*PiconPIP1;2, PiconPIP2;1, PiconPIP2;2, PiconPIP2;3,* and *PiconTIP1;1*) from eight families of *Pinus contorta* displaying source of variation, F-value and p-value. Significant sources of variation highlighted in bold.

**Source of Variation**	**F-value**	***p*-value**
PiconPIP1;2		
Family	27.45	**<0.001**
Drought	30.05	**<0.001**
Family × Drought	1.30	0.283
PiconPIP2;1		
Family	25.23	**<0.001**
Drought	4.47	**0.042**
Family × Drought	10.05	**<0.001**
PiconPIP2;2		
Family	22.70	**<0.001**
Drought	2.42	0.129
Family × Drought	12.45	**<0.001**
PiconPIP2;3		
Family	52.29	**<0.001**
Drought	0.28	0.603
Family × Drought	6.27	**<0.001**
PiconTIP1;1		
Family	9.08	**<0.001**
Drought	0.01	0.921
Family × Drought	6.70	**<0.001**

Table S3. Primer name and sequences for quantitative real-time PCR analysis of five aquaporins and two internal control genes.

Primer name	Primer sequences
Pine_ACT7	For: 5′- CCTTCCATCGTCCACAGAAA -3′<br>Rev: 5′- CCACCATCATCTCAAGCAAAC -3′
Pine_EF-1a	For: 5′- CTGCGGTCAAGAAGGGTAAA -3′<br>Rev: 5′- CTCGTGCATCAGAATCAGACA -3′
PinePIP1;2_qPCR	For: 5′- GGACTGGTAACACCTGTGTATTG -3′<br>Rev: 5′- CAGATGGCTCTTGGCCTAAAT -3′
PinePIP2;2_qPCR	For: 5′- GTCAGTGTCTCAAGTCTGTAGG -3′<br>Rev: 5′- ATTCTCCTCACTGTTGCTACTT -3′
PinePIP2;1_qPCR	For: 5′- AAAGTCCAGGGTGATGATGAG -3′<br>Rev: 5′- CTGCTGCTACTGTGACTACAA -3′
PinePIP2;3_qPCR	For: 5′- CAGGTGGACTTGTCTGTGTATC -3′<br>Rev: 5′- TTCTCTTCACCGGTTTCTCATC -3′
PineTIP1;1_qPCR	For: 5′- GGCGTTATCTACGAGCTGTTTA -3′<br>Rev: 5′- GCCCTGTAAGAAGCCACTTTA -3′

Table S4. Aquaporin gene and primer sequences for cloning five aquaporin genes.

Gene	Primer sequences
PiconPIP1;2	For: 5′- ATGGCGACTTTCTTGTTTCTTT -3′<br>Rev: 5′- CTAGGAGCGAGTTTTGAAGGG -3
PiconPIP2;1	For: 5′- ATGACAAAGGAAGAAAGGAAGGA -3′<br>Rev: 5′- TTACACATTTGCGTTGCTTCG -3′
PiconPIP2;2	For: 5′- ATGGCTAAGGAAGGTGGCAA -3′<br>Rev: 5′- GGGTTGCTGCGGAAGGAT -3′
PiconPIP2;3	For: 5′- ATGATAAATGAAGAAGGAGCTGTATCGG -3′<br>Rev: 5′- TTACGCATTAACGTTGCCTCGGAAC -3′
PiconTIP1;1	For: 5′- ATGCCGTTCGGAGGAATTGC -3′<br>Rev: 5′- TCAGTACTCGTTCGAGGGCAAC -3′

Table S5. Primer name and sequences for 3′ rapid amplification of cDNA ends (RACE).

Primer name	Primer sequences
3'-RACE-Q0	5′- CCAGTGAGCAGAGTGACG -3′
3'-RACE-Q1	5′- GAGGACTCGAGCTCAAGC -3′
3'-RACE-QT	5′- CCAGTGAGCAGAGTGACGAGGACTCG<br>AGCTCAAGCTTTTTTTTTTTTTTTTT -3′
*PiconPIP2;*3-GSP1	5′- CTGCCGCTGATCTCATTGAC -3′
PiconPIP2;3-GSP2	5′- TTGGTTTACTGCACTGCTGG -3′
*PiconPIP2;*1-GSP1	5′- AGAAGTTGAGCAGCAGGGAT -3′
PiconPIP2;1-GSP2	5′- ATGTGGCCAAGGACTACACA -3′

PiconPIP2;2-GSP1	5′- GAAGGAATTGGAGCAGCAGG -3′
PiconPIP2;2-GSP2	5′- GGGCATATCAATCCTGCTGT -3′
PiconPIP1;2-GSP1	5′- AGATGTTAACCTCGGTGCGA -3′
PiconPIP1;2-GSP2	5′- CACAGTCATGGGTGTCAAGG -3′
PiconTIP1;1-GSP1	5′- GGCATGGCTTTCGACAAACT -3′
PiconTIP1;1-GSP2	5′- GGCACATCACGCTTCTAAGG -3′

```
                                                                      H1
PiconPIP2;1   MAKEGGKEVEQQGYVAKDYTDPPPAALFDVGEFKLWAFYRAVIAEFIATLLFLYITVATV   60
PiconPIP2;2   MTKEERKELEQQGFPSKDYTDPPPAALIETSEFKLWSFYRALIAEFTATLLFLYITIATV   60
PiconPIP2;3   MINEEGAVSEQRGFVGKDYTDPPAADLIDTNEFKLWSFYRALIAEFTATLLFLYITVATV   60

                LA                 H2                  HB          LB
PiconPIP2;1   IGHKRNQTACGSVGLLGIAWAFGGMIFVLVYCTAGISGGHINPAVTFGLFLARKVSLPRA   120
PiconPIP2;2   IGHSRNSTNCGSVGVLGIAWSFGGMIFVLVYCTAGISGGHINPAVTFGLFLARKVSLPRA   120
PiconPIP2;3   IGYARTSTDCGSVGVLGIAWSFGGMIFILVYCTAGISGGHINPAVTFGLFLARKVSLPRA   120

                 H3                    LC                          H4
PiconPIP2;1   VLYMVAQCLGAICGCGLVKAFQKSYYDEYGGGANSVAHGYTKGVGLSAEIIGTFVLVYTV   180
PiconPIP2;2   ILYMIAQCLGAICGTGLVKAFQKSFYDRYGGGANYVHHGYTKGVGLAAEIIGTFVLVYTV   180
PiconPIP2;3   VLYMIAQCLGAICGTGLVKAFQKSFYDKYGGGANYVHPGYTKGVGLAAEIIGTFVLVYTV   180

                  LD                 H5                  LE      HE
PiconPIP2;1   FSATDPKRNARDSHIPVLAPLPIGFAVFMVHLATIPITGTGINPARSFGAAVIYGHQKSW   240
PiconPIP2;2   FSATDPKRSARDSHVPVLAPLPIGFAVFMVHLATIPITGTGINPARSFGAAVIYGRKQPW   240
PiconPIP2;3   FSATDPKRSARDSHVPVLAPLPIGFSVFMVHLATIPITGTGINPARSFGAAVTFGHKQSW   240

                         H6
PiconPIP2;1   DDQWIFWVGPFLGAAGAAAYHQYILRAGAIKALGSFRSNPHV   282
PiconPIP2;2   NDQWIFWVGPFVGAALASAYHQYILRAAAIKALGSFRSNANV   282
PiconPIP2;3   DDHWIFWVGPFVGAALAAAYHQYILRAAAIKALGSFRGNVNA   282
```

(**a**)

```
PiconPIP1;2   MEGKEEDVNLGANKYSERQPLGTAAQTQEKDYTEPGPAPLFEPGEFVSWSFYRAGIAEFM   60

                 H1               LA                  H2                 HB
PiconPIP1;2   ATFLFLYITILTVMGVKDATDICNGSVGIQGIAWAFGGMIFCLVYCTAGISGGHINPAVT   120

                 LB               H3                            LC
PiconPIP1;2   FGLFLARKLSLPRALLYMICQCLGAMCGAGVVKGFGPSQYEFNGGGANTVAHGYTKGDGL   180

                 H4                LD                 H5                LE  HE
PiconPIP1;2   GAEIVGTFVLVYTVFSATDAKRSARDSHVPILAPLPIGFAVFLVHLATIPITGTGINPAR   240

                                  H6
PiconPIP1;2   SLGAAIVFDSSHAWHDQWIFWVGPFIGAALAAFYHVIVIRAIPFKSRH   288
```

(**b**)

```
                                         H1                        LA
PiconTIP1;1   MPFGGIAVGRPEEATHPEALKAALAEGISTLIFVFAGEGSGMAFDKLTSDASTTPAGLVA   60

                 H2                HB         LB                  H3
PiconTIP1;1   VALAHALGLFVAVAVGANISGGHVNPAVTFGAFVGGHITLLRGILYWFAQLIGATVACLL   120

                LC                  H4                          LD        H5
PiconTIP1;1   LKFTTGGLSTSAFSLSSGVGVGNAVVFEIVMTFGLVYTVYATAVDPKKGNLGTIAPICIG   180

                         LE   HE                          H6
PiconTIP1;1   FIVGANILAGGAFDGASMNPARAFGPALVSWTWENHWIYWVGPLLGGGLAGVIYELFMIS   240

PiconTIP1;1   PEPTHQPLPSNEY   253
```

(**c**)

Figure S1. Protein sequence alignment of (**a**) PiconPIP2;1, PiconPIP2;2 and PiconPIP2;3; (**b**) PiconPIP1;2; and (**c**) PiconTIP1;1 sequence showing conserved six membrane-spanning helices (H1-H6): highlighted and italicized and five connecting loops (LA-LE). Amino acids at NPA domains: green; ar/R selectivity filters: red; Froger's P1–P5 residues: turquoise.
